# Persistent Pancytopenia as a Long-COVID Manifestation in a Patient with Adult-Onset Still’s Disease: A Case Report

**DOI:** 10.3390/medicina59071349

**Published:** 2023-07-23

**Authors:** Mattia Massimino, Francesco Salvatore Iaquinta, Saverio Naty, Francesco Andreozzi, Rosa Daniela Grembiale

**Affiliations:** 1 Department of Medical and Surgical Sciences, “Magna Græcia” University of Catanzaro, 88100 Catanzaro, Italy; mattia.massimino@hotmail.it (M.M.);; 2 Department of Health Sciences, “Magna Græcia” University of Catanzaro, 88100 Catanzaro, Italy; s.iaquinta1@gmail.com (F.S.I.); saverio_naty@yahoo.it (S.N.)

**Keywords:** COVID-19, pancytopenia, rheumatology, Still’s disease, adult-onset

## Abstract

*Background:* Adult-onset Still’s disease (AOSD) is a rare rheumatic inflammatory condition with an extremely heterogeneous clinical presentation and systemic impairment. Uncommon manifestations may be challenging to manage, especially in patients with previous severe acute SARS-CoV-2 infection. For the first time, we report the case of a patient affected by refractory AOSD presenting with severe pancytopenia as a long-COVID manifestation. The purpose of this case report is to illustrate the clinical presentation, diagnostic and therapeutic management of this unusual manifestation. Moreover, we examine the mechanisms that are potentially responsible for the onset of the pancytopenia observed in our patient. *Case presentation:* We describe the case of a 40-year-old male who presented with a history of fever for 2 years, arthralgia, maculopapular salmon-pink rash and a previous SARS-CoV-2 infection which required admission to intensive care. The patient’s laboratory results revealed elevated inflammatory markers levels (erythrocyte sedimentation rate and C-reactive protein), hyperferritinemia and severe pancytopenia that needed multiple transfusions. A diagnosis of AOSD was made based on clinical and laboratory presentation after excluding neoplastic, infectious and other rheumatic diseases. The previous empirical treatment was not adequate to control the condition; therefore, treatment with high-dose steroids, canakinumab and epoetin alfa was started and led to the resolution of the man’s symptoms and a reduction in inflammatory marker levels, whereas blood cell count remained stable without a need for further blood transfusions. The patient is currently under rheumatologic and hematologic follow-up every month. *Conclusions:* Neither AOSD nor SARS-CoV-2 infection usually manifests with pancytopenia, except in hemophagocytic syndrome or immunodeficient patients, respectively. Identifying the underlying etiology of pancytopenia is mandatory to establish a prompt treatment that generally resolves the disorder. However, in our case, all common causes of pancytopenia were excluded, suggesting a potential manifestation of the long-COVID syndrome. Despite the resolution of the acute infection and the remarkable treatment of AOSD, pancytopenia persists. Herein, we propose for refractory AOSD patients with previous SARS-CoV-2 infection a novel approach to the diagnosis and treatment of pancytopenia.

## 1. Introduction

Adult-onset Still’s disease (AOSD) is a rare, multi-systemic inflammatory condition of unknown etiology [1] probably triggered by environmental factors or microbes, including viruses that can facilitate or even initiate the process that leads to AOSD in genetically predisposed individuals [2]. The usual clinical presentation includes the triad of spiking fever, polyarthritis or arthralgias and salmon-pink macular or maculopapular evanescent rash, although manifestations can be extremely varied [3]. Other common symptoms, such as lymph node enlargement, hepatosplenomegaly, sore throat and pharyngitis, are often misdiagnosed. Laboratory findings frequently include hyperferritinemia, neutrophilic leukocytosis, anemia and thrombocytosis. AOSD is considered an archetype of auto-inflammatory disease; however, the pathogenesis remains unclear [1]. Due to difficult identification and the absence of specific diagnostic tests, AOSD is a diagnosis of exclusion, made through a set of validated criteria such as Yamaguchi’s classification criteria [4]. Therapeutic management is challenging, and many patients fail to respond or lose response to the first- and second-line therapy, identifying refractory AOSD [1].

SARS-CoV-2 is a new coronavirus that causes Coronavirus Disease 2019 (COVID-19) that typically presents with respiratory symptoms, including acute respiratory distress syndrome (ARDS) with high mortality [5]. However, several extrapulmonary manifestations (EPMs) have been reported, resulting in a heterogeneous clinical presentation; diarrhea, nausea/vomiting and abdominal pain can be onset symptoms, while neurologic, renal, cardiac and dermatological manifestations can delay diagnosis or aggravate the prognosis. SARS-CoV-2 has also been associated with hematological manifestations such as leukocytosis, lymphopenia, neutrophilia and thrombocytopenia [6].

We report a case of pancytopenia in a 40-year-old man with refractory AOSD as a long-COVID manifestation.

## 2. Case Presentation

In April 2021, a 40-year-old Caucasian male presented to the Internal Medicine Unit of the “Mater Domini” University Hospital in Catanzaro, Italy, with generalized joint pain and spiking high-grade fever (up to 39.7 °C). The patient denied smoking habits or substance abuse. The medical history presented a two-year-lasting fever of unknown origin, myalgia and multiple tender cervical and inguinal lymph nodes, but no hematological disorders. Although he was hospitalized several times in another medical center, the fine-needle aspiration cytology of the inguinal lymph node performed showed nonspecific lymphadenitis and, therefore, no specific diagnosis was made. As an empiric therapeutic approach, he was treated with broad-spectrum antibiotics, high-dose steroids with a prolonged taper of corticosteroids (cumulative dose in two years 19,905 mg) and methotrexate, which was suspended because of toxicity-related anemia and poor clinical control. In December 2020, during the COVID-19 wave in Italy, the patient was admitted to another hospital to continue the diagnostic and therapeutic course. At the time of admission, both the nasopharyngeal RT-PCR (reverse transcription polymerase chain reaction) swab and the serological test were negative for SARS-CoV-2. After almost two weeks of hospitalization, as the patient had contact with another COVID-19-positive patient, a nasopharyngeal swab was performed and tested positive. In the course of the infection, he developed tachypnea and desaturation; the chest computed tomography (CT) scan showed more than 75% ground-glass lung parenchymal opacities. Considering the clinical and radiological scenario, with severe bilateral interstitial pneumonia, the patient required admission to intensive care. Awake pronation and helmet continuous positive airway pressure (CPAP) were added to the therapy. However, despite the intensive therapeutic approach, the patient’s clinical conditions did not improve and a bilinear cytopenia (anemia and thrombocytopenia) occurred.

Consequently, to treat the severe form of COVID-19 in a patient with suspected AOSD, Anakinra was administered, with an improvement in the pneumological symptoms but no improvement in the hematological disorder being observed. Ten days after starting the drug, it was discontinued due to an adverse injection site reaction. Due to the new onset of bilinear cytopenia, bone marrow aspirate was performed to rule out myelodysplastic syndrome, and no abnormalities were found. On day 40, the patient tested negative for the nasopharyngeal swab for SARS-CoV-2.

**Figure 1:**
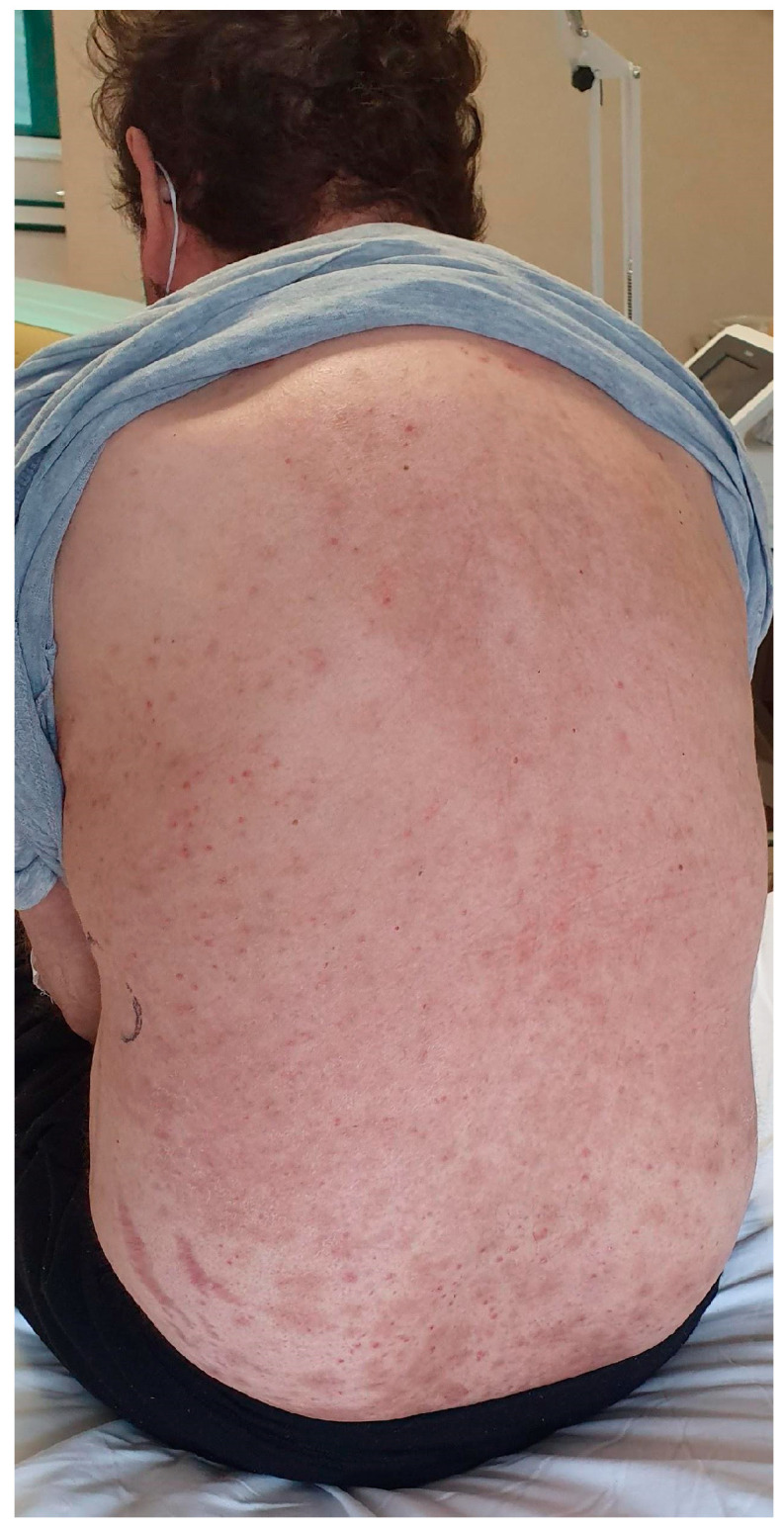
Evanescent “salmon-pink” skin rash on the trunk observed at the patient’s admission in April 2021.

In April 2021, before being admitted to our hospital, the patient underwent a nasopharyngeal RT-PCR swap for SARS-CoV-2 that was negative. Upon arrival at our hospital, the patient was suffering, pale, dyspnoeic and had a fever (39 °C) that tended to occur in the evening. A sore throat and a non-pruritic maculopapular salmon-pink rash on the back were observed (Figure 1).

Cervical lymphadenopathy, mild hepatosplenomegaly and bilateral swelling and tenderness of the metacarpophalangeal joints and wrists were present. A respiratory examination revealed bilateral pulmonary crackles. The pulse rate was 102 bpm with a non-pathological cardiovascular and neurological examination.

The patient’s laboratory results at admission revealed elevated levels of inflammatory markers, such as C-reactive protein (CRP), measured using an immunoturbidimetric method with an automated system (CardioPhase hsCRP, Milan, Italy); erythrocyte sedimentation rate (ESR), evaluated through microphotometrical capillary stopped-flow kinetic analysis (Roller 20 LC, Alifax, Padova, Italy); and ferritinemia dosed with an immunoturbidimetric assay (Roche Diagnostics, Indianapolis, IN, USA). The initial hematochemical and biochemical analysis also showed a low blood cell count (measured using an automated particle counter; ADVIA 120/2120 Haematology System; Siemens Healthcare Diagnostics, Italy) with pancytopenia. Lastly, vitamin B12 was also significantly elevated (Table 1).

**Figure 2:**
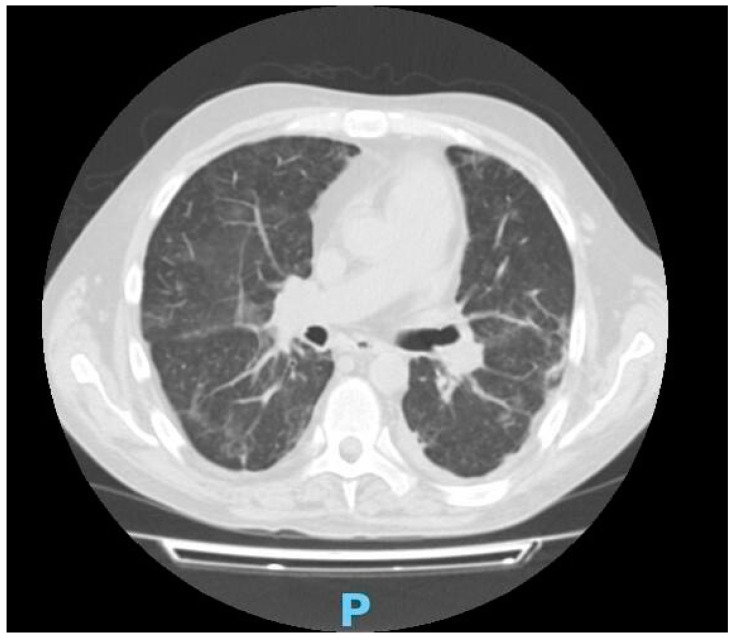
Axial CT image showing ground-glass opacities, peripheral septal thickening and minimal pericardial effusion; P: posterior.

Liver, thyroid and renal function tests and urinalysis were unremarkable. Procalcitonin and three blood culture sets taken during a 24 h period were negative. The peripheral blood smear did not show schistocytes, whereas tests for antinuclear antibodies (ANA), rheumatoid factor (RF) and anti-cyclic citrullinated peptide (ACPA) were negative. Complement C3 and C4 blood tests were in the normal range. Serological investigations showed that the patient had not previously been exposed to Mycobacterium tuberculosis, Brucella, Borrelia, Rickettsia, HIV, cytomegalovirus (CMV), HCV or leishmania, while testing for chlamydia, mycoplasma, Epstein–Barr virus (EBV) and HBsAb revealed previous infections. Chest computerized tomography scans reported peripheral septal thickening, bilateral ground-glass opacities and minimal pericardial effusion (Figure 2), and an ultrasound of the abdomen confirmed hepatosplenomegaly.

After excluding malignancies, infections and other rheumatic inflammatory conditions, the patient was diagnosed with AOSD according to Yamaguchi criteria [4] (Table 2).

During hospitalization, due to the sudden worsening of his general condition and the decline in his blood cell count, the patient needed multiple transfusions. A bone marrow biopsy was performed to exclude hemophagocytic lymphohistiocytosis (HLH) and intravenous treatment with methylprednisolone 60 mg daily and canakinumab 150 mg/mL every 8 weeks was started, resulting in a progressive improvement in symptoms and a decrease in the patient’s inflammatory index level. The biopsy showed a normal marrow architecture with effective erythropoiesis and granulocyte hyperplasia, but no signs of histiocytic activation. Despite the general improvement, the hematological disorder persisted, and the patient was evaluated for other potential causes of pancytopenia including infections, radiation therapy or chemotherapy, myelodysplastic syndrome, hemolytic anemia, megaloblastic anemia, connective tissue diseases, immunosuppressive medications and drug-induced bone marrow toxicity.

Due to the absence of other causes, we suspected a correlation with previous SARS-CoV-2 infection as a long-COVID manifestation, and therapy with epoetin alfa 20,000 iu/weekly was started.

At the following visits, laboratory findings showed steady hematological parameters (Table 1). Therefore, methylprednisolone was reduced to 24 mg/daily, continuing therapy with canakinumab 150 mg/mL every 8 weeks. The patient is currently symptom-free and under multidisciplinary follow-up every month including rheumatologic and hematologic visits, complete blood cell count and inflammatory markers. He also tested negative for the nasopharyngeal swap analysis for SARS-CoV-2 at 3 and 6 months.

## 3. Discussion

Our patient showed a severe hematological disorder, which is an unusual presentation of AOSD. The differential diagnosis may be challenging, as is the therapeutic management. In the present case, following extensive examinations, several causes of pancytopenia could be rejected (infections, radiation therapy or chemotherapy, myelodysplastic syndrome, hemolytic anemia, aplastic anemia, connective tissue diseases and/or immunosuppressive medications [7]). Macrocytic anemia has been considered one of the main differential diagnoses of pancytopenia. In this regard, macrocytic megaloblastic anemia was excluded through evaluation of MCV, peripheral blood smear analysis, vitamin B12 and B9 dosage. Hemolytic anemia was ruled out via a peripheral blood smear since no schistocytes were observed; moreover, the patient presented reticulocytes within the normal range, as well as total and fractioned bilirubin, transaminases and normal urinalysis. On the other hand, myelodysplastic syndrome, for which macrocytic anemia is the classic presentation, was excluded according to bone marrow biopsies and gene mutational analysis via next-generation sequencing (NGS) both at the herein-described and previous hospitalizations. Furthermore, due to neutropenia, thrombocytopenia, elevated ferritin levels and hepatosplenomegaly, HLH, a life-threatening disorder, was hypothesized; yet the bone marrow biopsy performed did not show histiocyte activation.

Since the beginning of the pandemic, millions of people have been infected with SARS-CoV-2, with the emergence of new complex clinical manifestations. In the year before the publication of this study, the persistence of symptoms after the resolution of acute infection was described as a novel condition named “long-COVID”, which describes signs and/or symptoms that continue for more than 12 weeks after acute COVID-19 and cannot be explained by an alternative diagnosis [8].

Due to the patient’s previous SARS-CoV-2 infection and its severe systemic involvement, we hypothesized a correlation with pancytopenia. Angiotensin-converting enzyme 2 receptors (ACE2 receptors), the primary target of spike proteins, are found in bone marrow at low levels [9] and, although pancytopenia is not a frequent complication of SARS-CoV-2 infection, recent evidence suggests that the virus could induce pancytopenia through different mechanisms, including cytokine storm syndrome or direct infection of myelocytes with consequent destruction of blood cells [10].

So far, few data are available on the characteristics of COVID-19-related pancytopenia. The few reported cases of COVID-19-induced pancytopenia mainly relate to acute infection in which the hematological disorder was self-limiting or improved with the resolution of the infection [11,12,13], but none of these concerned rheumatic diseases. Issa et al. [14] described the first case of persistent pancytopenia associated with SARS-CoV-2 bone marrow infiltration, suggesting the need to search for the virus via blood and bone marrow aspiration.

Usually, the management of pancytopenia depends on the underlying cause. However, for this new novel presentation, no evidence of proper treatment is available. In the case of three lines of cytopenia, along with the treatment of the underlying cause, standard therapy consists of supportive care including red blood cell and platelet transfusions. The use of granulocyte-colony-stimulating factor (G-CSF) in patients with SARS-CoV-2 infection is controversial due to the evidence of an increased risk of mortality and morbidity [15]; this is the reason why it was not administrated to our patient. Nevertheless, in our case, the patient responded to and remained stable after aggressive immunosuppressive treatment and epoetin alfa administration. However, the adequate treatment of long-COVID hematological manifestations is far from established.

Long-COVID syndrome has been reported globally and is typically associated with pulmonary (usually interstitial pneumonia, diffuse alveolar fibrosis, microhemorrhages) [16,17], cardiovascular (cardiac arrhythmias, ischemic and non-ischemic cardiomyopathy, pericarditis, myocarditis, pulmonary embolism and ictus) [18,19], nervous system (sleepiness, fatigue, brain fog, headache, impaired memory and concentration) [20] and hematological complications (lymphopenia, neutropenia, thrombocytopenia, thrombosis and anemia) [21]. Pancytopenia is not a disease but a laboratory term describing the reduction in all three types of blood cells. Several cases of pancytopenia as a manifestation of long-COVID disease have been reported in the medical literature [12,13,14,22,23].

In this study, for the first time, we report the case of a man with refractory AOSD presenting with severe pancytopenia of unknown origin. Given the absence of other possible explanations for cytopenia and a past SARS-CoV-2 infection, long-COVID syndrome was considered the main cause of the hematological disorder. According to the literature on pancytopenia complicating AOSD and/or SARS-CoV-2 infection, we hypothesize that the virus led to direct damage to the bone marrow, resulting in the hematological features of our patient. Nevertheless, further studies on the underlying mechanisms of bone marrow suppression in patients with AOSD, and, broadly, in patients with rheumatic diseases, are required to support this relationship.

## 4. Conclusions

As the SARS-CoV-2 pandemic continues to spread, further hematologic manifestations may arise. We propose that patients with inflammatory rheumatic diseases who experience severe acute SARS-CoV-2 infection and long-COVID syndrome should be evaluated by a multidisciplinary team; a diagnosis of pancytopenia origin may not require a bone marrow biopsy, except when HLH is suspected, and the treatment should include aggressive immunosuppressive therapy along with stimulation of the bone marrow and supportive care if needed.

## Figures and Tables

**Table 1:** Initial laboratory examinations and parameters at 3 and 6 months at “Mater Domini” University Hospital of Catanzaro, Italy.

Hematochemical and Biochemical Parameters				
Tests	Admission	3 Months	6 Months	Normal Ranges
**Complete blood count**				
Red blood cell count (10^6^/μL)	2.94	2.24	2.67	4.2–5.4
Hemoglobin (g/dL)	8.7	10.5	9.4	12–16
White blood cells (10^3^/μL)	2.94	3.63	2.65	5.2–12.4
Neutrophils (10^3^/μL)	1.16	2.06	1.71	1.9–8
Lymphocytes (10^3^/μL)	1.46	1.18	0.62	0.9–5.2
Monocytes (10^3^/μL)	0.22	0.27	0.20	0.16–1
Eosinophils (10^3^/μL)	0.01	0.01	0.10	0–0.8
Basophils (10^3^/μL)	0.02	0.03	0.01	0–0.2
Platelets (10^3^/μL)	48	52	39	130–400
Mean corpuscular volume (fL)	89.6	93.9	95.6	81–99
Mean cell hemoglobin (pg)	29.3	37.2	35.1	27–31
Mean corpuscular hemoglobin concentration (g/dL)	32.7	37.3	33.3	31–37
Ferritin (ng/mL)	3744	1561	-	30–400
C-reactive protein (mg/L)	250	25.3	<3.23	0–5
Erythrocyte sedimentation rate (mm/h)	42	4	10	<30
Fibrinogen (mg/dL)	624	324	301	200–400
Vitamin B12 (pg/mL)	1336	1497	-	191–663

**Table 2:** Yamaguchi classification criteria for AOSD (sensitivity: 96.2%; specificity: 92.1%).

**Major criteria:**	Fever > 39 °C lasting at least 1 week Arthralgia or arthritis lasting >2 weeks Typical non-pruritic salmon-pink skin rash Leukocytosis 10,000/mm^3^ with granulocytes 80%
**Minor criteria:**	Sore throat Lymph node enlargement Hepatomegaly or splenomegaly Abnormal liver function tests Negative antinuclear antibody (ANA) and rheumatoid factor tests
**Exclusion criteria:**	Infections Malignancy Other rheumatic disorders
**For diagnosis of AOSD: ≥5 criteria, of which at least 2 are major and no exclusion criteria.**	

## Data Availability

The data presented in this study are available on request from the corresponding author. The data are not publicly available due to privacy restrictions.
